# How Are You Feeling? Interpretation of Emotions through Facial Expressions of People Wearing Different Personal Protective Equipment: An Observational Study

**DOI:** 10.3390/nursrep12040075

**Published:** 2022-10-17

**Authors:** José Luis Díaz-Agea, María José Pujalte-Jesús, Vanessa Arizo-Luque, Juan Antonio García-Méndez, Isabel López-Chicheri-García, Andrés Rojo-Rojo

**Affiliations:** Faculty of Nursing, Universidad Católica de Murcia (UCAM), Av. de los Jerónimos, 135, Guadalupe, 30107 Murcia, Spain

**Keywords:** coronavirus infections, expressed emotion, health personnel, protective devices, professional burnout

## Abstract

(1) Background: The perception of others’ emotions based on non-verbal cues, such as facial expressions, is fundamental for interpersonal communication and mutual support. Using personal protection equipment (PPE) in a work environment during the SAR-CoV-2 pandemic challenged health professionals’ ability to recognise emotions and expressions while wearing PPE. The working hypothesis of this study was that the increased limitation of facial visibility, due to the use of a personal protective device, would interfere with the perception of basic emotions in the participants. (2) Methods: Through a cross-sectional descriptive study, the present research aimed to analyse the identification of four basic emotions (happiness; sadness; fear/surprise; and disgust/anger) through three types of PPE (FFP2 respirator, protective overall and powered air-purifying respirator (PAPR)), by using 32 photographs. The study was conducted using volunteer participants who met the inclusion criteria (individuals older than 13 without cognitive limitations). Participants had to recognise the emotions of actors in photographs that were randomly displayed in an online form. (3) Results: In general, the 690 participants better recognised happiness and fear, independently of the PPE utilised. Women could better identify different emotions, along with university graduates and young and middle-aged adults. Emotional identification was at its worst when the participants wore protective overalls (5.42 ± 1.22), followed by the PAPR (5.83 ± 1.38); the best scores were obtained using the FFP2 masks (6.57 ± 1.20). Sadness was the least recognised emotion, regardless of age. (4) Conclusions: The personal protective devices interfere in the recognition of emotions, with the protective overalls having the greatest impact, and the FFP2 mask the least. The emotions that were best recognised were happiness and fear/surprise, while the least recognised emotion was sadness. Women were better at identifying emotions, as well as participants with higher education, and young and middle-aged adults.

## 1. Introduction

During the coronavirus (SAR-CoV-2) pandemic, health professionals worldwide were exposed to a high risk of infection. This risk was greater at the beginning of the pandemic, when the mechanisms of transmission were not completely clear. Throughout the months in which the virus spread worldwide, health services adapted to this situation with protection guidelines for workers, to decrease the risk of infection. These guidelines included every type of PPE, which, in each case, eliminated the visual perception of essential parts of the worker’s face. The use of surgical masks, goggles or a face shield, gowns and respirators for specific procedures (i.e., N95 or FFP2 standard or equivalent) [1] were recommended. In some cases (especially in intensive care units), more complex equipment was and is being used, such as powered air-purifying respirators (PAPRs) [2,3]. All this equipment can reduce non-verbal communication based on facial expressions [4,5] among the professionals who use them, and between health professionals and patients.

Health professionals were strongly affected, not only due to exposure to physical dangers but also to stress, which negatively impacted their mental health [6,7,8,9,10]. For health professionals, adequate communication skills were shown to be a fundamental aspect in preventing Burnout Syndrome [11]. The communication of emotions, and their detection by the rest of the work team, could lead to the early activation of mutual support behaviours in situations of continued stress. These emotions can be shared verbally or can be detected consciously or unconsciously [12] through non-verbal cues, principally through facial expressions. However, due to the measures that restrict personal contact in healthcare environments, and the physical barrier presented by wearing PPE, the detection of other’s emotions could be altered, especially with respect to the interpretation of facial expressions through this equipment [13].

The objective of the present study was to analyse the interpretation of emotions manifested through facial expressions when wearing different types of facial protection equipment, and to identify the emotions that are easier or more difficult to interpret when wearing different types of PPE. The study’s main hypothesis was that the PPE, which hides part of the face, impedes the correct interpretation of the emotions of the individuals wearing them.

Emotions are generated as reactions to important events (those that are irrelevant do not produce emotion) [14]. Emotions result in motivational states, activate the body for action and produce recognisable facial expressions. Although emotions generate feelings, they differ from feelings as they have a direct connection with the world (emotions are expressed and can be recognised by others [15]). That is the social dimension of emotion, as it can be expressed verbally or non-verbally and recognised by another human being. This aspect is essential in communication and interaction between people [16]. 

Another critical aspect is the involuntary character of emotion: to put it simply, emotions are automatic. Emotions have an adaptive value, which is linked to the expression of a feeling in response to an important event. If a human being recognises the expression of emotion in another person, she or he can then have an idea about how the other is feeling and adjust her or his behaviour accordingly (if someone is sad, we can console or provide support, for example). In the healthcare context, the interpretation of the patient’s emotions, and that of co-workers, is important. On many occasions, it has implications for the activation of support behaviours and mutual care, especially in a context of work overload, such as that found presently in healthcare settings [17,18].

Facial recognition of emotion is the primary way to know the emotional state of an individual and consequently activate an appropriate affective response [19]. Until the present, authors have discussed whether or not the recognition of emotions is innate or universal. Many prototypical universal emotions have been described. The classic work by Paul Ekman [20,21], in which he described six emotions that are present in every human culture (anger, fear, disgust, surprise, sadness and happiness), has been the basis of almost all of the research conducted with respect to the facial recognition of emotions. At the time of writing, specific software programmes have been developed, so that computers to recognise the emotional patterns in human faces [22].

Very recently, up to 16 recognisable facial expressions were associated with emotions, which can occur in any social context in any part of the world [23]; however, it seems that only four universal basic emotions can be clearly and immediately recognised, without any context to provide us with more information [24]. The latter study postulated that facial signals evolve from biologically basic minimal information to socially specific complex information. Accordingly, the signalling dynamic allows the discrimination of four categories of emotions (and not six, which has prevailed until now). In line with this, fear, surprise, anger and disgust are frequently misread. Although facial expressions of happiness and sadness are different from start to finish, both fear and surprise share a base signal before they are produced: the eyes are fully opened. Likewise, disgust/anger have common facial signals, such as a wrinkled nose, which are observed right before the emotion is shown.

For this reason, the present work focused on the analysis of these four prototypical basic units of emotion (happiness, sadness, fear/surprise and disgust/anger), following the pattern proposed by Ekman in 1970 [20], discerning facial expressions with images, and adapting it to the context of personal protection equipment. The working hypothesis of this study was that the increased limitation in the visibility of the face due to the use of a personal protective device would interfere with the perception of basic emotions in the participants.

## 2. Materials and Methods

A cross-sectional and descriptive observational study was designed to evaluate photographs by volunteers [20,25,26]. We followed STROBE reporting guidelines (see Appendix A).

### 2.1. Participants, Procedure and Data Collection

The study was conducted using volunteer participants who met the inclusion criteria (individuals older than 13 without cognitive limitations). Participants had to recognise the emotions of actors in photographs that were randomly displayed in an online form. Thirty-two high-resolution pictures of two actors, a Caucasian man and woman, were taken. In these pictures, they appeared both without PPE and wearing different PPEs that partially covered their faces. The PPEs chosen were an FFP2 mask, protective overalls (PO)/ body, mask, protection goggles and, finally, a powered air-purifying respirator (PAPR). The actors gave their interpretations of four different universal basic emotions (happiness, sadness, fear/surprise and disgust/anger), which are not easily mistaken [24].

**Figure 1:**
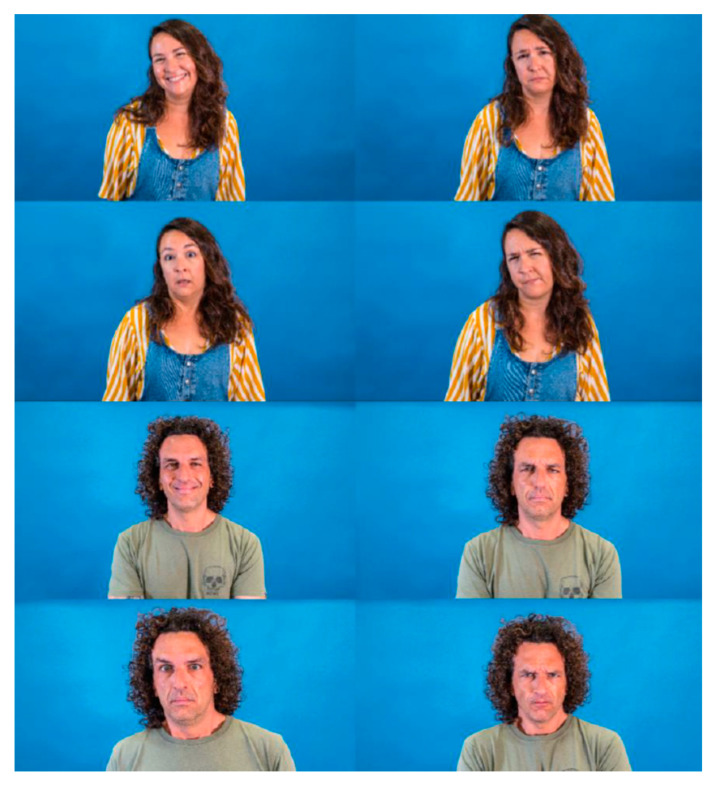
Photographs of the four emotions by an actress and an actor without personal protection equipment (from left to right: happy; sad; fear/surprise; and disgust/anger). *Both actors have agreed to display their images for this research.

**Figure 2:**
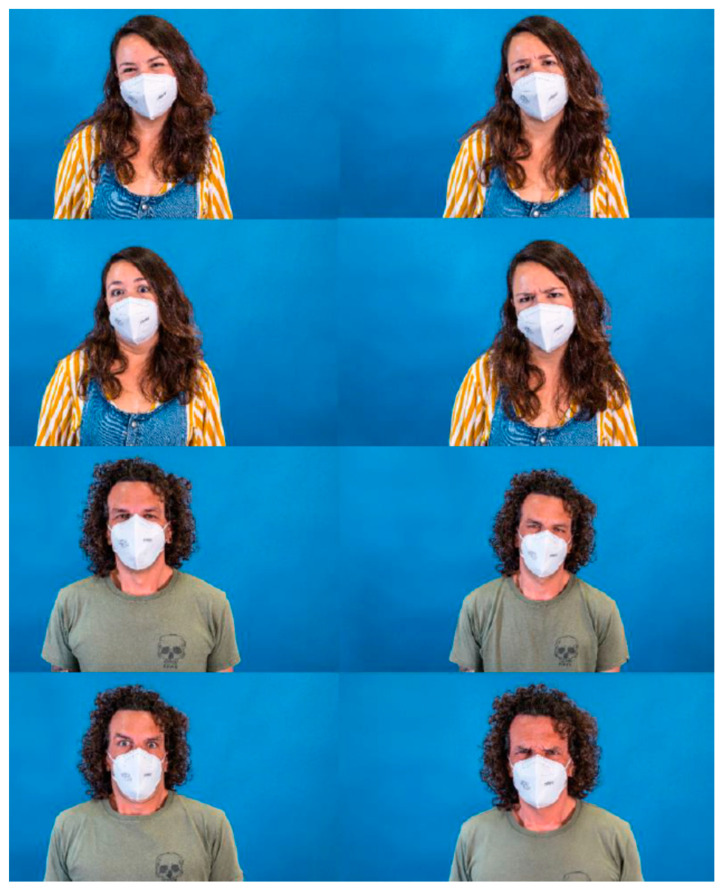
Photographs of the four emotions by an actress and an actor with an FFP2 mask (from left to right: happy; sad; fear/surprise; and disgust/anger).

**Figure 3:**
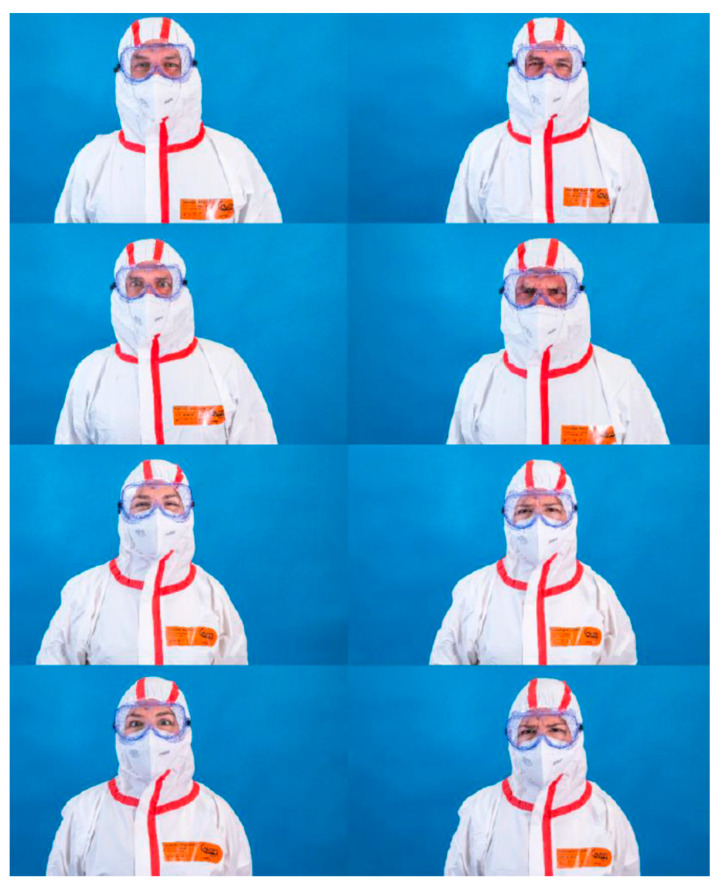
Photographs of the four emotions by an actress and an actor wearing protective overalls (PO)/body, mask and protection goggles (from left to right: happy; sad; fear/surprise; and disgust/anger).

**Figure 4:**
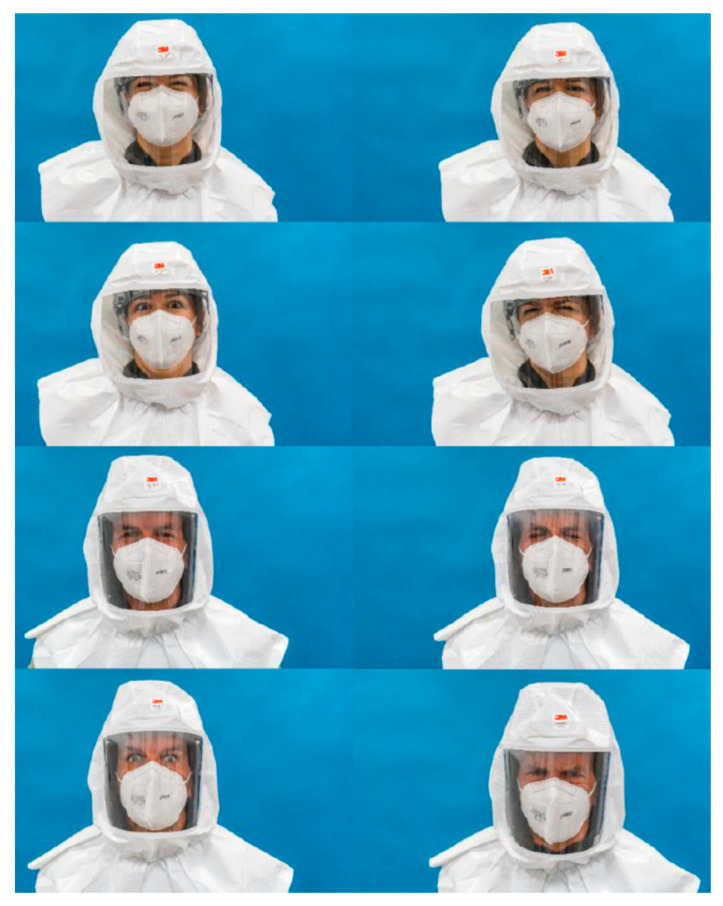
Photographs of the four emotions by an actress and an actor wearing a powered air-purifying respirator-PAPR and an FFP2 mask (from left to right: happy; sad; fear/surprise; and disgust/anger).

For the interpretation/acting of the emotions, the actors had a period of adaptation and used the prototypical images present in similar, validated studies as the gold standard [26]. Figure 1, Figure 2, Figure 3 and Figure 4 show the actor’s photographs according to the type of PPE/emotion.

The 32 photographs were inserted into a Google Forms form (Google Forms^®^), which, aside from explaining the objective of the study, collected sociodemographic information and asked the participants for their consent (the form is available at https://forms.gle/rVxCBWSsWNr49udEA accessed on 7 July 2021). The photographs randomly appeared in the form each time the questionnaire was opened. The form encouraged the participants to identify the emotion transmitted by the actor/actress from the five response options, which coincided with the four emotions studied (happiness, sadness, fear/surprise and disgust/anger), and an optional answer (I am not sure). At the end of the questionnaire, the following questions were asked: (1) Which device have you had more difficulties identifying emotions with? and (2) Which part of the face do you consider to be more relevant for interpreting others’ emotions?

For the selection of the participants, we took into account the innate human ability to recognise primary forms of affective expression (any person can recognise the emotions of others) [27]. The questionnaire was distributed through social networks and email groups throughout Spain. The study was conducted using volunteer participants who met the inclusion criteria (individuals older than 13 without cognitive limitations who responded to the online form within a specific period of 3 months, from July to September 2021). Participants in the study were chosen by convenience (no statistical sampling was used). The study was approved by the Ethics Committee of the Catholic University of Murcia (Spain), dated 27 July 2020 (reference number: CE072002).

### 2.2. Analysis

The data collection was conducted through the online questionnaire and is detailed in the following section. The sociodemographic variables collected were gender (male/female), age (in years), city/country of origin and level of education (basic: mandatory secondary education; medium: high school/practical training; high: university).

Student’s *t*-test was utilised to analyse the existence of statistically significant differences between the dichotomous variable means (sex and nationality (Spanish/other)), and an ANOVA test was used in those with three and more categories. Post-hoc test used were Bonferroni with equal variances and Tahmane in different ones. The categorical variables were described as frequencies and percentages (%). To compare the data, three variables were created that measured the correct identification of each emotion in the photographs, with a point given to each response that coincided with the emotion expressed:

(1) Score per emotion, with a maximum score of 8.
(2) Score by type of PPE, with a maximum score of 8.
(3) Total score per individual, achieved by adding all the responses given with the correct identification, with a maximum score of 32.

The processing and analysis of the data was conducted with the statistical package IBM SPSS^®^ v.22.0 for Windows.

## 3. Results

From the 702 responses obtained, seven were excluded as participant consent was not provided, and five were excluded were due to the participants being underage. Thus, the responses from 690 individuals were analysed (*n* = 690). The mean age of the participants was 41 years (SD 12.97), of which 473 (69%) were women and 665 (96%) were Spanish. As for the level of education, 79% had a university degree, 16% had a high school/practical training degree and 5% had primary education. Once the age data were analysed, the participants were divided into four age groups: younger than 18; 19–40 (young adults); 41–60 (middle-aged adults); and older than 60. According to these groupings, the sample was composed of 10 individuals younger than 18 years old; 310 were aged between 19 and 40 years old; 306 were aged between 41–60 years old; and 56 were older than 60.

After looking at the images, the participants best recognised the emotions of happiness and fear, followed by anger, regardless of the PPE utilised (Table 1). The emotion of sadness was the most difficult to recognise, regardless of age. The participants were unsure when trying to identify it, and frequently confused it with anger (*p* = 0.047 [*t*-Student with Bonferroni correction]).

According to gender, statistically significant differences were found in the identification of all the emotions except for happiness, with women obtaining a better mean value (24.95 ± 3.41) than men (23.93 ± 3.6) (Table 2). In addition, women recognised the emotions better than men when the actor/actress was wearing a PAPR (*p* = 0.013) or an FFP2 mask (*p* < 0.001) (Table 3).

The results did not show statistically significant differences between the Spanish nationals and foreign participants concerning the recognition of emotions as a function of PPE utilised.

According to age groups, statistically significant differences were found concerning emotions, except for sadness (happiness: F = 5.48 *p*-value = 0.001; sadness: F = 2.83 *p*-value = 0.038; fear: F = 2.65 *p*-value = 0.048; anger: F = 6.67 *p*-value < 0.001). In general, the 19–40 years group recognised emotions better than those in the 41–60 years group (mean difference: 1.01 *p* = 0.002) and those older than 60 (mean difference: 1.38 *p* = 0.037). More specifically, the 19–40 years group recognised anger better than those in the 41–60 years group (mean difference: 0.43 *p*= 0.001) and those older than 60 (mean difference: 0.60 *p* = 0.013), and happiness (mean difference: 0.22 *p* = 0.049) better than those in the the 41–60 years group. According to age groups, regarding the type of PPE (PAPR: F = 5.55 *p*-value = 0.001; PO: F = 1.44 *p*-value = 0.231; FFP2: F = 2.34 *p*-value = 0.072; no PPE: F = 4.26 *p*-value = 0.005), the 19–40 years group recognised emotions better with the PAPR than those in the 41–60 years group (mean difference: 0.38 *p* = 0.003) and those older than 60 (mean difference: 0.59 *p* = 0.017); they also recognised emotions better without PPE than those in the 41–60 years group (mean difference: 0.19 *p* = 0.041). 

Concerning the level of education, the results showed statistically significant differences in recognition of emotions when the actors wore the PAPR (F = 5.21 *p* = 0.006), the PO (F = 3.39 *p* = 0.034), the FFP2 mask (F = 3.04 *p* = 0.048), and when PPE was not used (F = 11.28 *p* < 0.001). In general, participants with a higher level of education were better at recognising emotions than those with lower education levels. Participants with a university education were more capable of recognising all the emotions, with significant differences found between the participants with a basic level of education when the actors wore the PAPR (mean difference: 0.60 *p* = 0.028), the PO (mean difference: 0.53 *p* = 0.028), and when a PPE was not used (mean difference: 0.0.47 *p* = 0.006). When compared to those with a medium level of education (high school), participants with higher education (university) were better at recognising emotions when the actors did not wear any PPE (mean difference: 0.37 *p* < 0.001). The group of participants aged 18 years or younger did not show different results from the rest of the participants in the recognition of emotions.

When the participants were asked about the part of the face that was most significant in the expression of emotions, 80% indicated that the eyes, eyebrows and the degree of eye widening were the most important, and 20% considered the mouth more important (smile, grimaces, etc.) (PAPR: F = 5.78 *p*-value = 0.003; PO: F = 1.59 *p*-value = 0.204; FFP2: F = 4.89 *p*-value = 0.008; no PPE: F = 0.88 *p*-value = 0.417). Both groups obtained similar scores when PPE was absent (7.89 vs. 7.79). However, when PPE was present, the former obtained better scores in recognition of happiness (mean difference: 0.52 *p* < 0.001), wearing the PAPR (mean difference: 0.37 *p* = 0.019) and with the FFP2 (mean difference: 0.36 *p* = 0.017), when compared to the latter.

Participants indicated that the most challenging situation to recognise emotions was when the actors wore the PAPR. However, the lowest scores were obtained when the actors wore the PO (5.42 ± 1.22), followed by the PAPR (5.83 ± 1.38). The best scores were obtained when the FFP2 mask images were shown (6.57 ± 1.20). When the PPE was absent, the mean score obtained in recognition of emotions was 6.82 ± 0.91, from a maximum of 8 points.

## 4. Discussion

The main findings from the present study show that the ability to interpret emotions through facial expressions is reduced when individuals wear different types of PPE. Thus, it is expected that, even without conducting research, we could therefore deduce that any obstacle that reduces the visibility of facial expressions could, in theory, influence the recognition of such expressions by others. In the present work, we studied how this influence could be observed in a large sample of individuals who viewed images of actors wearing different types of PPE.

The different variables considered when collecting and interpreting the data gave us a broad view of the types of emotions that were easy or more difficult to recognise, and the influence of age and gender when interpreting others’ emotions. The level of education also influenced how the participants recognised the emotions of the actors. Specifically, we analysed the impact of the type of PPE when participants were trying to recognise the emotions of individuals wearing them.

Different contemporary studies [28,29] described how physical barriers that hide the face interfere in the interpretation of verbal and non-verbal cues in human interaction. In our study, the types of emotions that were easy to recognise were happiness and fear/surprise. This was the case in each kind of photograph evaluated. Another study found that the expression of the eyes was not as crucial as the mouth in recognition of happiness [30], but a more recent investigation found that wearing a mask may increase the recruitment of the eyes during smiling, activating the orbicularis oculi when smiling with a mask [31]. In our study, the eyes and the area surrounding them was the only facial element involved in making expressions, excepting those images in which the actors did not wear any PPE. In our study, the emotion that was more difficult to recognise was sadness, which was confused with anger on many occasions, as has been previously found [32]. The fact that sadness was the most poorly recognised emotion in people wearing PPE has implications for clinical practice, especially concerning mutual support between professionals subjected to an elevated stress level.

The pandemic has led to a significant increase in stress and work overload for health professionals [7,33,34]. For the activation of support behaviours between co-workers [35], the negative emotions must be expressed by the person who is feeling exhausted, stressed or sad, or be detected by others. The difficulty in the recognition of sadness by the rest of the work team could impede the adequate development of mutual support behaviours. This could be important in initiating support behaviours by co-workers and in individuals who do not explicitly speak about their emotions. Moreover, it has been stated that the impact of the mask on interpreting emotions could even be more decisive in natural settings, because, in everyday interactions, we invest less attention and time in looking for emotional cues on others’ faces [32].

Another interesting finding in our study was that women were able to identify emotions better than men, especially when the actress/actor wore a PAPR or FFP2 mask, and we can therefore discuss emotional intelligence, defined as the ability of individuals to recognise their own emotions and that of others [36]. Although it could be thought of as stereotypical, many studies [37,38,39,40,41] argue that women are better at recognising emotions in others since they have been socialised to be more empathetic and perceptive. The field of neuroscience has demonstrated [42,43] the existence of larger areas in woman’s brains that are dedicated to the processing of emotions. These differences could also be because of the socialization process. Furthermore, other studies have demonstrated that gender differences in emotional intelligence decrease substantially or disappear when age is considered a mediator and that age accounted for a higher percentage of the variance in emotional intelligence [44]. In Spain, 72% of the health professionals currently employed are women, and 84% of the registered nurses in Spain are also women [45]. This could also have implications for the ease of emotional recognition, despite the use of PPE by primarily female health professionals, and could provide a gendered perspective to the problem of emotion recognition and the putting into practice of mutual support behaviours.

With regards to the age of the participants, the group that was able to better recognise emotions were those in the 19–40-year range, both in general (without PPE) and in the presence of the PAPR. In older age, the ability to recognise emotions seems to decrease [46], evidenced by the decreased stimulation observed in older people when facing emotional situations, which makes sense in the context of our study. As for the level of education, the individuals with a high level of education were better at recognising emotions (especially university graduates) in every situation. 

Regarding the type of PPE, more difficulties were found in the recognition of emotions with the PAPR at the subjective level (when participants were asked about the device with which they believed they would have a greater difficulty). However, the lowest scores were found with the PO, and the highest with the FFP2 mask. These results show that greater exposure of areas of the face leads to a better interpretation of emotions by others.

As for the areas considered fundamental for the recognition of emotions by participants, the eyes obtained the highest scores. This idea is in agreement with other studies, which investigated how the eyes and mouth were the elements that were most involved in the facial recognition of others’ emotions, from sadness and fear (trust in the eyes) to disgust and happiness (mouth) [30].

One of the limitations of this study was that it was restricted to Spanish speakers. The external validity of the study is perhaps limited for this reason. Statistical sampling was also not carried out, and the sample was chosen by convenience. However, all analyses were conducted with a sample of 690 participants and a significance level of 0.05; therefore, even for a small effect size (d = 0.2) the power of each test would be 0.9995.

## 5. Conclusions

The results from this study provide evidence that PPE (which is commonly used by health professionals in times of high risk of infections) interferes in the recognition of emotions, with the PO interfering the most and the FFP2 mask the least. The emotions that were best recognised were happiness and fear/surprise, while the least recognised emotion was sadness. Women were better at identifying emotions, as well as participants with higher education, and young and middle-aged adults.

### Relevance for Clinical Practice

While it is true that the recognition of co-workers’ emotions could be affected by the use of PPE, especially with regard to negative emotions, we believe that the interaction with patients wearing masks should be taken into account. As an implication for clinical practice, we can add that patients’ feelings are often not verbalised and are perceived by professionals through non-verbal cues. This perception could be limited using PPE. We propose the establishment of protocols for detecting emotions, which include open questions that show the professional’s interest in patients’ emotions. This aspect is fundamental in the development of the work of healthcare professionals [47].

Health professionals must receive training related to emotional intelligence and verbal expression of emotions, so that systems of mutual support between co-workers can be activated as soon as possible.

Detecting emotions in the professional area of nursing is fundamental, especially concerning situations of stressm such as those experienced during the pandemic, which necessacitated, more than ever, the activation of empathetic attitudes and behaviours.

The use of PPE should be adapted to each situation and should be based on scientific evidence, to allow for adequate interpersonal communication.

Employers and health institutions must be aware of the factors that impede the adequate detection of emotions and must implement actions to prevent negative emotional states and to detect them as early as possible, especially when the personal protection equipment artificially alters normal non-verbal communication.

## Figures and Tables

**Table 1:** The percentage of agreement (success) in identifying the emotions: happiness, fear/surprise, anger/disgust and sadness in the 32 photographs (*n* = 690).

Type of PPE †		Emotions (% Agreement)			
		Happiness	Fear/Surprise	Anger/Disgust	Sadness
PAPR †	Women	80.6%	98.4%	49.1%	33.7%
	Men	84.5%	91.9%	88.1%	55.6%
PO ‡	Women	98.5%	69.6%	69.2%	32.0%
	Men	89.8%	92.4%	91.5%	15.8%
FFP2	Women	99.1%	87.0%	89.3%	58.9%
	Men	70.7%	93.3%,	93.4%	46.3%
No PPE §	Women	100%	96.9%	94.5%	85.0%
	Men	99.1%	92.4%	97.2%	88.0%

† Powered air-purifying respirator. ‡ Protective Overalls. § Personal protection equipment.

**Table 2:** Success scores of the participants according to gender and emotion (mean [standard deviation], t-score [degrees of freedom], *p*-value and mean difference) (*n* = 690).

	Gender	Mean (SD)	t-Score (df)	*p*-Value †	MD
Total score (maximum 32)	Women	24.94 (3.41)	3.6 (688) *	<0.001	1.018
	Men	23.93 (3.60)			
Score: Happiness (maximum 8)	Women	7.21 (1.03)	−0.02 (688) *	0.988	−0.001
	Men	7.22 (1.04)			
Score: Sadness (maximum 8)	Women	3.57 (1.59)	3.4 (688) *	0.001	0.439
	Men	3.13(1.54)			
Score: Fear (maximum 8)	Women	7.13(1.15)	2.0 (688) *	0.44	0.196
	Men	6.93 (1.24)			
Score: Anger (maximum 8)	Women	6.67 (1.28)	3.5 (365) **	<0.001	0.419
	Men	6.25 (1.51)			

* Assuming equal variances. ** Assuming unequal variances. † *t*-student test.

**Table 3:** Success scores of the participants according to gender and type of Personal protection equipment (maximum 8 points) (mean [standard deviation], t-score [degrees of freedom], *p*-value and mean difference) (*n* = 690).

	Gender	Mean (SD)	t-Score (df)	*p*-Value †	MD
Score: PAPR ‡	Women	5.92 (1.37)	2.5 (688) *	0.013	0.281
	Men	5.64 (1.38)			
Score: PO Φ	Women	5.47 (1.19)	1.7 (688) *	0.087	0.172
	Men	5.29 (1.27)			
Score: FFP2	Women	6.71 (1.13)	4.4 (372) **	<0.001	0.447
	Men	6.27 (1.30)			
Score: no PPE §	Women	6.84 (0.91)	1.6 (688) *	0.115	0.118
	Men	6.72 (0.90)			

* Assuming equal variances. ** Assuming unequal variances. † *t*-student test. ‡ Powered air-purifying respirator. Φ Protective Overalls. § Personal protection equipment.

## Data Availability

The data are available to those who wish to access them by sending an email to the corresponding authors.

## Appendix A. STROBE Reporting Guidelines Checklist

**Table A1:** STROBE Statement—checklist of items that should be included in reports of observational studies.

	Item No.	Recommendation	Page No.	Line with Relevant Text from Manuscript
Title and abstract	1	(a) Indicate the study’s design with a commonly used term in the title or the abstract	1	4
		(b) Provide in the abstract an informative and balanced summary of what was done and what was found	1	11–31
Introduction				
Background/rationale	2	Explain the scientific background and rationale for the investigation being reported	1–2	41–110
Objectives	3	State specific objectives, including any prespecified hypotheses	3	110–113
Methods				
Study design	4	Present key elements of study design early in the paper	3	115–117
Setting	5	Describe the setting, locations, and relevant dates, including periods of recruitment, exposure, follow-up, and data collection	8	119–170
Participants	6	(a) Cross-sectional study—Give the eligibility criteria, and the sources and methods of selection of participants	8	147–149
Variables	7	Clearly define all outcomes, exposures, predictors, potential confounders, and effect modifiers. Give diagnostic criteria, if applicable	8	165–171
Data sources/ measurement	8 *	For each variable of interest, give sources of data and details of methods of assessment (measurement). Describe comparability of assessment methods if there is more than one group	8	171–176
Bias	9	Describe any efforts to address potential sources of bias	7	149–153
Study size	10	Explain how the study size was arrived at	8	198
Quantitative variables	11	Explain how quantitative variables were handled in the analyses. If applicable, describe which groupings were chosen and why	8	171–190
Statistical methods	12	(a) Describe all statistical methods, including those used to control for confounding	8	171–190
		(b) Describe any methods used to examine subgroups and interactions	8	157–164
		(c) Explain how missing data were addressed	-	
		(d) If applicable, describe analytical methods taking account of sampling strategy	-	
		(e) Describe any sensitivity analyses	-	
Results				
Participants	13 *	(a) Report numbers of individuals at each stage of study—eg numbers potentially eligible, examined for eligibility, confirmed eligible, included in the study, completing follow-up, and analysed	8	198–205
		(b) Give reasons for non-participation at each stage	-	
		(c) Consider use of a flow diagram	-	
Descriptive data	14 *	(a) Give characteristics of study participants (eg demographic, clinical, social) and information on exposures and potential confounders	8	198–205
		(b) Indicate number of participants with missing data for each variable of interest	-	
Outcome data	15 *	Cross-sectional study—Report numbers of outcome events or summary measures	9–10	Table 1, Table 2 and Table 3
Main results	16	(a) Give unadjusted estimates and, if applicable, confounder-adjusted estimates and their precision (e.g., 95% confidence interval). Make clear which confounders were adjusted for and why they were included	9–10–11	
		(b) Report category boundaries when continuous variables were categorized	8	181–185
		(c) If relevant, consider translating estimates of relative risk into absolute risk for a meaningful time period	-	
Other analyses	17	Report other analyses done—eg analyses of subgroups and interactions, and sensitivity analyses	-	
Discussion				
Key results	18	Summarise key results with reference to study objectives	12, 13	272–353
Limitations	19	Discuss limitations of the study, taking into account sources of potential bias or imprecision. Discuss both direction and magnitude of any potential bias	13	349–353
Interpretation	20	Give a cautious overall interpretation of results considering objectives, limitations, multiplicity of analyses, results from similar studies, and other relevant evidence	11–12	
Generalisability	21	Discuss the generalisability (external validity) of the study results	13	355–385
Other information				
Funding	22	Give the source of funding and the role of the funders for the present study and, if applicable, for the original study on which the present article is based	14	392–393

* Give information separately for cases and controls in case-control studies and, if applicable, for exposed and unexposed groups in cohort and cross-sectional studies.
